# Microclimate exerts greater control over litter decomposition and enzyme activity than litter quality in an alpine forest-tundra ecotone

**DOI:** 10.1038/s41598-018-33186-4

**Published:** 2018-10-09

**Authors:** Yamei Chen, Yang Liu, Jian Zhang, Wanqin Yang, Runlian He, Changchun Deng

**Affiliations:** 1 Long-term Research Station of Alpine Forest Ecosystems, Institute of Ecology & Forestry, Sichuan Agricultural University, Chengdu, 611130 China; 2 Collaborative Innovation Centre of Ecological Security in the Upper Reaches of the Yangtze River, Chengdu, 611130 China

## Abstract

Plant litter decomposition is an important biogeochemical process in terrestrial ecosystems. Although climate and substrate quality controls over litter decomposition are reasonably well understood, their impacts on lignocellulose degradation and lignocellulolytic enzymes remain elusive. Here, the decomposition of three leaf litters derived from *Salix paraplesia* (SP), *Deyeuxia scabrescens* (DS), and *Ajuga ovalifolia* (AO), was studied across an alpine forest-tundra ecotone during one snow-covered season with the objective of distinguishing between the effects of microclimate and litter quality on litter decomposition rates and lignocellulolytic enzymes. The results showed that both microclimate and litter quality affected lignocellulose degradation rates and lignocellulolytic enzyme activities; however, microclimate factors had the greater effects. Interestingly, freeze-thaw cycles and moisture were the predominant factors explaining the variations in decomposition rate and enzyme activities. Higher cellulose degradation rates were associated with higher cellulose concentrations. Cellulolytic enzymes had a greater effect on litter decomposition than did ligninolytic enzymes at the early decomposition stage. Litter decomposition and enzyme activities should be given more attention under global climate change, as the direction and magnitude of changes in microclimate factors and litter quality could strongly influence the nutrient cycling and energy fluxes of alpine ecosystems.

## Introduction

Litter decomposition is a key process in carbon (C) and nutrient cycling and is controlled by three main factors: climate, litter quality and decomposing organisms^1,2^. Plant leaf litter can contain considerable amounts of lignin (15–40%), cellulose and hemicelluloses (10–50%)^2^ and polyphenols^3^. In general, the depolymerisation of cellulose is a hydrolytic process involving three classes of cellulolytic (hydrolytic) enzymes: β−1,4-exoglucanase, endo-1,4-β-glucanase (EG), and β−1,4-glucosidase (βG)^4,5^. Lignin degradation occurs through oxidative reactions involving a complex set of ligninolytic (oxidative) enzymes, including laccase (Lac), manganese peroxidase (MnP), and lignin peroxidase (LiP)^6^. Extracellular enzymes are directly influenced by temperature and moisture and are released into the environment by microbial secretion and microbial cell lysis^7–9^, which are regulated by litter type^10,11^, temperature and moisture^12^.

Climate change can strongly impact abiotic and biotic factors in alpine ecosystems^13^. Average temperatures have increased globally in the last century^14,15^ and have been accompanied by variation in precipitation and snowpack patterns^16^. In the long term, there could be an upward shift of alpine plants, tree line advance^17^, shrub expansion in the alpine zone^16,18^, and graminoid expansion to alpine forb-dominated vegetation^19^. Previous studies have investigated the effects of climate change on litter decomposition and microbial community composition and activity along an elevational gradient, which comprises a suite of highly auto-correlated characteristics (i.e., vegetation, litter, soil microbiology, and snow cover)^20–22^. For example, litter mass loss is affected by litter type and decreases with elevation in the Italian Alps^23^. In addition, Berger *et al*.^20^ found that elevation retarded mass loss and associated C release of beech litter during the first year only and those of decaying pine litter for longer periods in the Austrian Alps. The dehydrogenase activity in alpine and subalpine soils^24^ and the cellulase and xylanase activities in the Austrian Central Alps^25^ decreased with increasing elevation. Another study reported that soil enzymes (i.e., β-glucosidase, N-acetylglucosaminidase, acid phosphatase, and leucine aminopeptidase) were higher at lower elevations along an elevational gradient from mixed coniferous forest to alpine tundra on Changbai Mountain, China^26^. However, there continues to be a lack of attention paid to the degradation of cellulose, lignin and polyphenols, which are the main macromolecular compounds in leaf litter, or to the lignocellulolytic enzymes^27^.

The alpine forest-tundra ecotone is the transition from coniferous forests to shrublands and alpine meadows^28^, so over short distances along the vegetation gradient, there are differences in microclimate and microbial community composition. Previous studies found substantial litter decomposition in the winter in alpine ecosystems^29–32^ that was attributed to physical destruction caused by freeze-thaw cycles, microbial activity, and hydraulic leaching as well as their integrated effects^26^. Furthermore, mass loss and cellulose and lignin degradation in the litter of some woody plants increased with increasing elevation^31^. However, the decomposition of other litter types (i.e., herb and graminoid litter) and enzyme activities remain to be studied. We hypothesized that 1) microclimate factors exerts greater control over litter decomposition and enzyme activity than does litter quality and 2) cellulolytic enzymes have a greater effect on litter decomposition than do ligninolytic enzymes at the early stage of litter decomposition. To test these hypotheses, litter decomposition and ligninolytic and cellulolytic enzymes were investigated along an elevational gradient (in coniferous forest, treeline and meadow ecosystems) across an alpine forest-tundra ecotone. Three representative types of leaf litter were chosen in this study: *Salix paraplesia* (SP), a deciduous shrub; *Deyeuxia scabrescens* (DS), a graminoid species; and *Ajuga ovalifolia* (AO), a forb species. The initial lignin and cellulose contents were found to differ significantly among these species. Our objective was to distinguish the effects of microclimate and litter quality (i.e., C, nitrogen (N), phosphorus (P), lignin, cellulose and phenols) on the rates of litter decomposition and the activities of ligninolytic and cellulolytic enzymes during one snow-covered season. Whether the activities of ligninolytic and cellulolytic enzymes influence litter decomposition was also evaluated.

## Materials and Methods

### Site description

Our study was conducted at Zhegu Mountain, an important riparian river area located at the eastern edge of the Tibetan Plateau in Li County, Sichuan Province, China (31°51′428′′N, 102°41′230′′E). The mountain ranges from 3200 to 4800 m a.s.l. and exhibits remarkable vertical zonality. From the valley to the hilltops, the area is composed of mixed coniferous–broadleaf forest, dark coniferous forest, alpine shrubland, and alpine meadow, with an alpine desert above 4500 m a.s.l. The alpine tree line, which is located around the upper elevational boundary of the coniferous forest, is approximately 4000 m a.s.l. The dominant plant species in the coniferous forest are *Abies faxoniana* and *Rhododendron taliense*, and the alpine shrubland is dominated by *Rosa omeiensis*, *Berberis silva-taroucana*, and others. The dominant herbaceous species of the alpine shrubland include *Epilobium angustifolium*, *Deyeuxia scabrescens*, and *Gentiana scabra*, and the alpine meadow is dominated by *Ajuga ovalifolia*, *Festuca wallichanica*, *Polygonum paleaceum*, *Pedicularis roylei*, and others. During the winter, there is a period of snow cover of up to 6 to 7 months, from October to April^31^. The soils in the coniferous forest and shrubland and in the alpine meadow are classified as Cambisols and a Histosol (World Reference Base taxonomy). The annual mean temperature and precipitation are approximately 2.9 °C and 870 mm, respectively, and the relative air humidity and atmospheric pressure are approximately 74% and 1000 hPa.

### Experimental design

**Figure 1:**
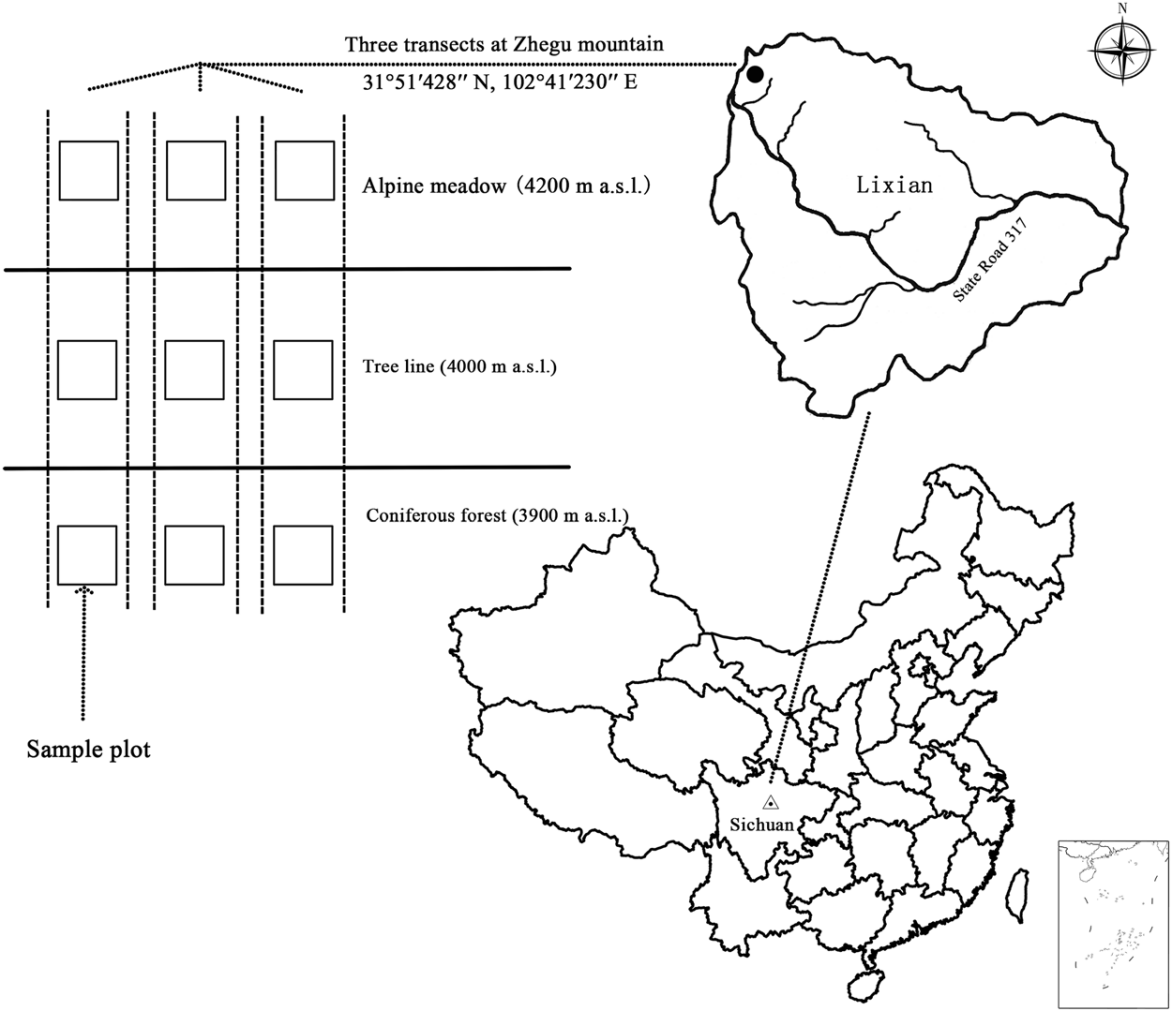
Diagram of the experimental layout of litterbags in the selected permanent sample plots across an alpine forest-tundra ecotone on the eastern Tibetan Plateau. The map of study area in China was generated with ARCGIS (version 10.2, ESRI, Redlands, CA, USA, http://desktop.arcgis.com/en/arcmap). The map and the diagram of the sample plots were assembled using PHOTOSHOP software (version 6.0; Adobe Systems, Mountain View, CA, https://www.adobe.com).

Three 50-m-wide transects, which were separated by more than 1 km, were set perpendicular to the contour line in the forest-tundra ecotone^31^. Along these three transects, three permanent sample plots (20 × 20 m) had been previously established in the coniferous forest (3900 m a.s.l.), alpine shrubland (4000 m a.s.l.), and alpine meadow (4200 m a.s.l.) (Fig. 1). Freshly abscised leaves of SP, DS, and AO were collected in October, when most of the litter fall occurs. After the removal of contaminating debris, the leaf litter was oven-dried at 65 °C for 48 h and mixed to provide a homogeneous sample. Portions of 10.00 g of leaf material were enclosed in nylon net bags (20 × 20 cm, surface-layer mesh of 1.0 mm and ground-layer mesh of 0.5 mm). Three bags for each species were used to determine the initial litter quality, and 54 litterbags (three vegetation types × three plots × three species × two litterbags) were prepared for the study (Fig. 1). Litterbags of each species were placed on top of the litter layer in sample plots in the coniferous forest, tree line, and alpine meadow on 30 October 2012, and the litterbags were placed at least 5 cm apart. The temperatures during litter decomposition were automatically monitored every 3 h using thermometers (iButton DS1921-F5, Maxim/Dallas Semiconductor, Sunnyvale, CA, USA) in the litterbags. The snow cover thickness was manually monitored every month. Six bags of each litter type were collected from each plot on 30 April 2013. Three were cleaned to measure the litter mass loss and litter quality, and the other three bags were used to determine the enzyme activity and moisture content. A subsample (approximately 60% of fresh weight) of litter from each bag was stored at 4 °C for enzyme extraction, and the remaining litter in the three bags was oven-dried at 65 °C to measure the moisture content.

### Chemical analyses

The C, N and P concentrations were determined as described by Lu^33^. The C concentration was determined using the dichromate oxidation-sulphate-ferrous titration method, in which 0.2000 g of the sub-samples were acid-digested with a solution of 8 mL of H_2_SO_4_ (98%) and 3 mL of H_2_O_2_ at 190 °C for 30 min. The digested solution was then transferred to a 100-mL volumetric flask, quantified, filtered, and stored to measure the N and P concentrations^30^, which were determined by the Kjeldahl method and molybdenum blue colorimetry, respectively.

The lignin and cellulose concentrations were determined using the acid detergent–lignin method^34^, with some modifications by He *et al*.^35^. One g of oven-dried and ground sub-samples was transferred to digestion tubes and suspended in a solution of H_2_SO_4_ (1.0 M) and cetyltrimethylammonium bromide (CTAB; 20 g L-1; 80 mL). The tubes were heated at 169 °C for 1 h, and after cooling, they were transferred to a sand core funnel (50 mL, G3 specification) and washed with acetone until the solution obtained through the suction filtration was clean. After oven drying at 170 °C for 1 h, the sample and tube were weighed together and designated W1. The sample was subsequently soaked for more than 3 h in an H_2_SO_4_ solution (72%), subjected to suction filtration and washed with acetone, as described above. The sample was then oven-dried at 170 °C for 1 h. The sample and tube were weighed together and designated W2, and both were then placed in a muffle furnace (Box Furnace; Lindberg/Blue M, Asheville, NC, USA) at 550 °C for 3 h and weighed after cooling (designated as W3). The cellulose and lignin concentrations (g·kg^−1^) were calculated as follows:

1 $${\rm{Cellulose}}\,{\rm{concentration}}=1000\times ({\rm{W}}1-{\rm{W}}2)$$

2 $${\rm{Lignin}}\,{\rm{concentration}}=1000\times ({\rm{W}}2-{\rm{W}}3)$$

Based on the oxidation–reduction principle, the total phenol concentration was determined using the Folin–Ciocalteu method^36^.

### Enzyme extraction and assays

Enzymes were extracted according to the methods described by Criquet *et al*.^37^ with minor modifications. First, 4 to 9 g of freshly powdered litter (<0.5 mm) were extracted overnight in 15 mL of 0.1 M CaCl_2_ solution with 0.05% Tween 80 and 0.40 g of polyvinylpolypyrrolidone at 4 °C. The suspension was centrifuged at 12,000 × g and 4 °C for 20 min, and the supernatant was subsequently dialysed for 48 h at 4 °C in 14-kDa molecular mass cut-off cellulose dialysis tubing against frequently exchanged 2 mM bis-Tris(bis[2-hydroxyethyl]imino-tris[hydroxymethyl] methane) buffer, pH 6.0. Extracts that had been boiled for 15 min served as controls for enzyme activity, except MnP activity for which the reaction mixture without Mn served as a control. Units of enzyme activities are calculated based on the mass of remaining C and expressed as nmol min^−1^ per g of C.

Unless otherwise indicated, all enzyme activities described below were analysed at the optimal pH value and temperature. Activity assay reaction mixtures of Lac, MnP, and LiP were allowed to proceed for 5 min at 30 °C^38^. Using syringaldazine as a substrate, Lac activity was measured according to the methods described by Criquet *et al*.^37^. The reaction mixture contained 2.5 mL of 0.1 M phosphate buffer (pH 5.7) and 0.1 mL of 5 mM syringaldazine solution. The rate of oxidation of syringaldazine to quinone was measured at 525 nm (ε = 65000 M^−1^ cm^−1^).

The MnP activity was measured according to the methods developed by Arora *et al*.^39^ and Fujii *et al*.^38^. Using phenol red as a substrate, the reaction mixture contained 2.0 mL of a sodium succinate buffer (50 mM) (pH 4.5), 2.0 mL of sodium lactate (50 mM), 0.8 mL of Mn sulphate (0.1 mM) (or an equimolar amount of EDTA for the control), 1.4 mL of phenol red (0.1 mM), 0.8 mL of H_2_O_2_ (50 mM), 2.0 mL of albumin (0.1%), and 1.0 mL of enzyme extract, and the reaction was initiated by adding H_2_O_2_. A 2-mL aliquot of the reaction mixture was removed and added to 40 mL of 5 M NaOH. The oxidation rate of phenol red was measured at 610 nm (ε = 4460 M^−1^ cm^−1^).

The LiP activity was measured according to the methods described by Arora *et al*.^39^ and Fujii *et al*.^38^. Using Azure B as the substrate, the reaction mixture contained 0.5 mL of a sodium tartrate buffer (50 mM) (pH 3.0), 0.5 mL of Azure B (32 mM), 0.5 mL of H_2_O_2_ (100 mM), and 0.5 mL of enzyme extract, and the reaction was initiated by adding H_2_O_2_. The oxidation rate of Azure B was measured at 651 nm (ε = 48800 M^−1^ cm^−1^).

The EG activity was determined by measuring the release of reducing sugars from appropriate substrates. The EG activity was measured according to methods described by Criquet^40^. The reaction mixture, which contained 0.2 mL of enzyme extract and 0.6 mL of 50 mM sodium acetate buffer (pH 6.0) containing 2% carboxymethylcellulose, was incubated for 1 h at 50 °C.

The βG activity was measured according to the methods described by Valášková *et al*.^41^. The reaction mixture contained 0.16 mL of 1.2 mM *p*-nitrophenyl-β-D-glucoside in 50 mM sodium acetate buffer (pH 5.0) and 0.04 mL of enzyme extract. The reaction mixtures were incubated at 40 °C for 40 min. The reaction was stopped by the addition of 0.1 mL of 0.5 M sodium carbonate, and the absorbance was read at 400 nm. The enzyme activity was calculated using the molar extinction coefficient of *p*-nitrophenol (11,600 M^−1^ cm^−1^).

Using birchwood xylan as the substrate^42^, the EX activity was measured by incubating 0.2 mL of the enzyme extract with 0.6 mL of 50 mM sodium acetate buffer (pH 6.0) containing 2% birchwood xylan for 1 h at 50 °C. The amount of reducing sugars was determined using the dinitrosalicylic acid method^43^.

### Calculations and statistics

Calculations of litter dry mass loss (*L*), and the release (*R*_*i*_) of C, cellulose, lignin and total phenol at the end of the snow-covered season were performed as follows:

3 $$L=100\times \frac{({{\rm{M}}}_{{\rm{0}}}-{{\rm{M}}}_{{\rm{i}}})}{{{\rm{M}}}_{{\rm{0}}}}$$

4 $$Ri( \% )={\rm{100}}\times \frac{({{\rm{M}}}_{{\rm{0}}}{{\rm{C}}}_{{\rm{0}}}-{{\rm{M}}}_{{\rm{i}}}{{\rm{C}}}_{{\rm{i}}})}{{{\rm{M}}}_{{\rm{0}}}{{\rm{C}}}_{{\rm{0}}}}$$

where M_0_ and M_i_ are the dry masses of the initial and remaining litter in the litterbags, respectively, and C_0_ and C_i_ are the concentration (g·kg^–1^) of C, cellulose, lignin and total phenol in the initial and remaining litter, respectively. To characterise the temperature parameters, we calculated the average temperature (AT), frequency of the freeze-thaw cycle (FFTC), positive accumulated temperature (PAT) and negative accumulated temperature (NAT) from the monitored temperature data^44^. The FFTC was calculated using the following definitions: a decrease to a temperature below 0 °C for at least 3 h followed by an increase to a temperature above 0 °C for at least 3 h or an increase to a temperature above 0 °C for at least 3 h followed by a decrease to a temperature below 0 °C for at least 3 h^45^. Moreover, the PAT and NAT were calculated as the sum of the average daily temperatures above/below 0 °C during the decomposition stage, respectively. The average thickness of the snow cover (ATSC) in different vegetation types was also calculated.

First, a two-way analysis of variance (ANOVA) was used to evaluate the effects of vegetation type, litter type, and their interaction on overall litter decomposition and enzyme activities in the litter. We also used non-metric multidimensional scaling (NMDS) with Bray-Curtis distance analysis to visualise the effects of vegetation type and litter type on overall litter decomposition and enzyme activities in the litter. One-way ANOVAs were conducted for initial litter quality. In all cases, more than two groups were examined, and least significant difference (LSD) tests were conducted to compare the associated group means. We performed a redundancy analysis (RDA) to determine which initial litter quality and environmental factors were related to the overall litter decomposition and enzyme activities and to determine the effect of enzyme activities on litter decomposition. When we conducted Monte Carlo permutation (n = 499), the “Summarise Effects of Expl. Variables” method was used to first individually determine the effect of each variable on the dependent variable and then to find the forces (independent variable) driving the dependent variable by adding new variables based on the order of explanation. We used variation partitioning analysis (VPA) to a) compare the effects of litter quality and environmental factors on litter decomposition and ligninolytic and cellulolytic enzyme activities and b) compare the effects of ligninolytic enzymes and cellulolytic enzyme activities on litter decomposition. Two-way ANOVAs, one-way ANOVAs and LSD tests were performed using SPSS Statistics for Windows (version 21.0). NMDS was performed with the vegan package in R software for Windows (version R 3.0.1), and RDA and VPA were performed using CANONCO software for Windows (version 5. 0).

## Results

### Microclimatic factors and initial litter quality

**Figure 2:**
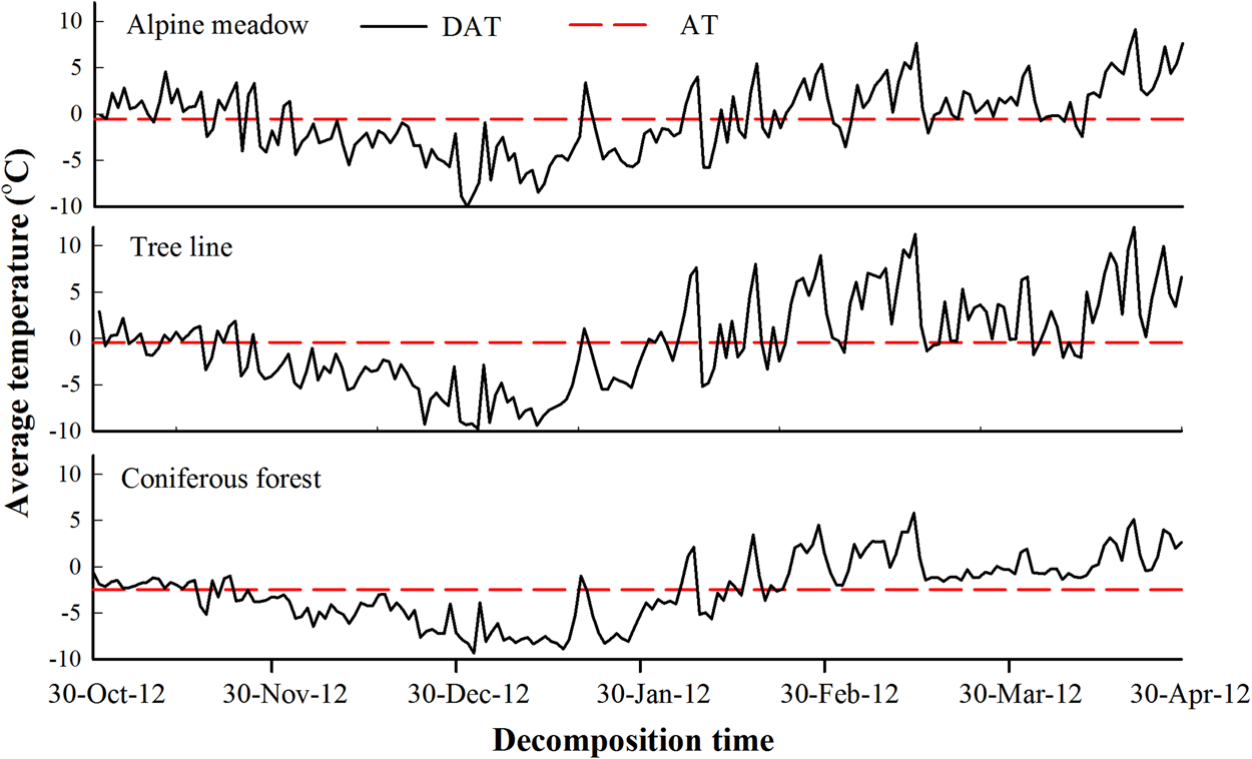
Dynamics of the average temperature in ambient leaf litter in the coniferous forest, at the tree line and in the meadow of the alpine forest-tundra ecotone from 30 October 2012 to 30 April 2013. DAT: daily average temperature; AT: average temperature.

The average daily temperature fluctuation was greater at the tree line and in the alpine meadow than in the coniferous forest, as shown in Fig. 2. The AT and PAT in winter for litter decomposition decreased across elevations at the alpine forest-tundra ecotone in the order tree line > alpine meadow > coniferous forest (Table 1). The ATSC decreased in the order coniferous forest (7.2 cm) > tree line (6.4 cm) > alpine meadow (1.1 cm), and the frequency of the freeze-thaw cycle (FFTC) was highest in the alpine meadow (327) followed by at the tree line (270) and in the coniferous forest (135) (Table 1). The average moisture content of the leaf litter was highest in the coniferous forest followed by in the alpine meadow and at the tree line (Table 1). The initial litter C concentration, P concentration and lignin concentration of SP were higher than those of DS and AO, and the lignin:N ratio of SP (9.24) was higher than that of DS (4.88). The initial litter total phenol and cellulose concentrations of DS were lower than those of SP and higher than those of AO (Table 2).

**Table 1:** Temperature parameters, average thickness of the snow cover and average moisture content of leaf litter undergoing decomposition during the snow-covered season in the coniferous forest, at the tree line, and in meadow sites in the alpine forest-tundra ecotone.

Vegetation type	AT (°C)	FFTC (times)	PAT (°C)	NAT (°C)	ATSC (cm)	AMC (%)
Coniferous forest	−2.5	135	86.5	−541.1	7.2	71.3
Tree line	−0.4	270	312.7	−393.1	6.4	26.4
Alpine meadow	−0.5	327	223.4	−323.1	1.1	37.0

AT, average temperature; FFTC, frequency of the freeze-thaw cycle; PAT, positive accumulated temperature; NAT, negative accumulated temperature; ATSC, average thickness of the snow cover; AMC, average moisture content.

**Table 2:** Initial quality of *Salix paraplesia* (SP), *Deyeuxia scabrescens* (DS), and *Ajuga ovalifolia* (AO) leaf litters (means ± SD, n = 3).

Litter type	C (g·kg^−1^)	N (g·kg^−1^)	P (g·kg^−1^)	C/N	C/P	Cellulose (g·kg^−1^)	Lignin (g·kg^−1^)	Lignin/N	TPh (g·kg^−1^)
SP	488.60 (13.80)^a^	18.6 (1.18)^a^	3.77 (0.05)^a^	26.34 (1.85)^a^	129.65 (4.02)^c^	75.07 (28.53)^c^	171.20 (50.37)^a^	9.24 (2.74)^a^	23.50 (0.13)^a^
DS	451.07 (16.21)^b^	12.95 (0.10)^a^	1.55 (0.01)^b^	34.85 (1.52)^a^	290.58 (12.70)^b^	285.18 (10.34)^a^	3.10 (6.20)^b^	4.88 (0.51)^b^	12.88 (0.53)^b^
AO	409.36 (2.87)^c^	13.47 (4.73)^a^	1.14 (0.04)^c^	32.63 (9.57)^a^	360.81 (14.71)^a^	186.30 (11.52)^b^	73.41 (0.95)^b^	5.84 (1.67)^ab^	22.51 (0.89)^a^

C, carbon concentration; N, nitrogen concentration; P, phosphorus concentration; TPh, total phenol concentration. Different lowercase letters indicate significant differences (P < 0.05) in the same variable among different species.

### Mass loss and element release

**Figure 3:**
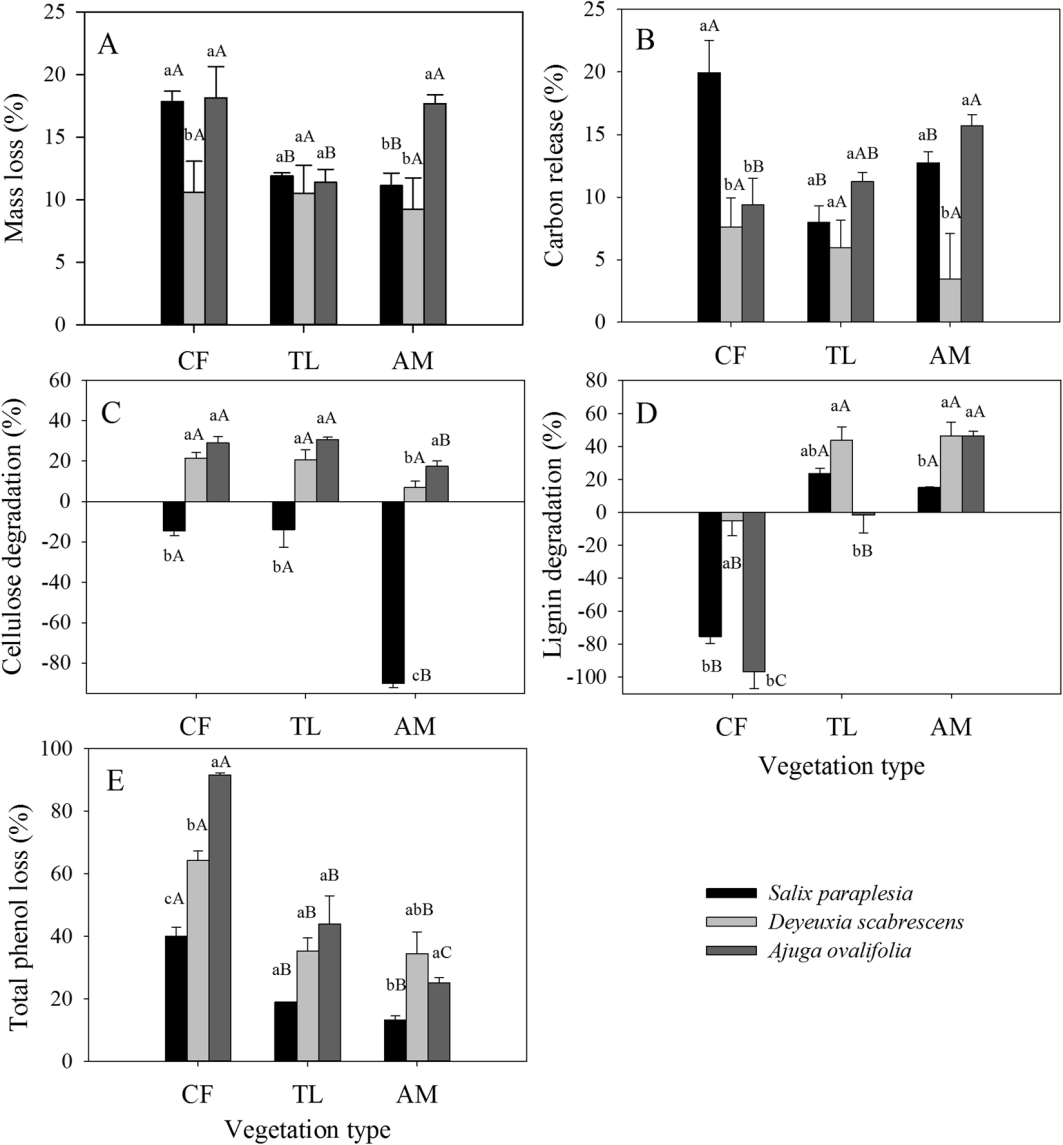
Mass loss and element release (%) of *Salix paraplesia* (SP), *Deyeuxia scabrescens* (DS), and *Ajuga ovalifolia* (AO) leaf litter. AM, alpine meadow; TL, tree line; CF, coniferous forest. Different lowercase letters indicate significant differences (P < 0.05) among different litter types within the same vegetation type, and different uppercase letters indicate significant differences (P < 0.05) among vegetation types within the same litter type. The data are the means ± SE (n = 3).

After a winter of decomposition, the mass loss and C release of SP were higher in the coniferous forest than in the alpine meadow and at the tree line, whereas the mass loss and C release of DS did not vary significantly with elevation (Fig. 3A and B). The cellulose degradation of SP and AO in the alpine meadow (−90% and 14%) was lower than that in the coniferous forest (−15% and 29%) or tree line (−14% and 30%), and the cellulose degradation of DS and AO was much higher than that of SP (Fig. 3C). In contrast, the lignin degradation of the three species in the coniferous forest (−5% to −97%) was much lower than that in the tree line (−2% to 43%) or alpine meadow (15% to 46%) (Fig. 3D). The total phenol loss of the three species was almost two times higher in the coniferous forest than in the tree line or alpine meadow (Fig. 3E).

### Ligninolytic and cellulolytic enzyme activities

**Figure 4:**
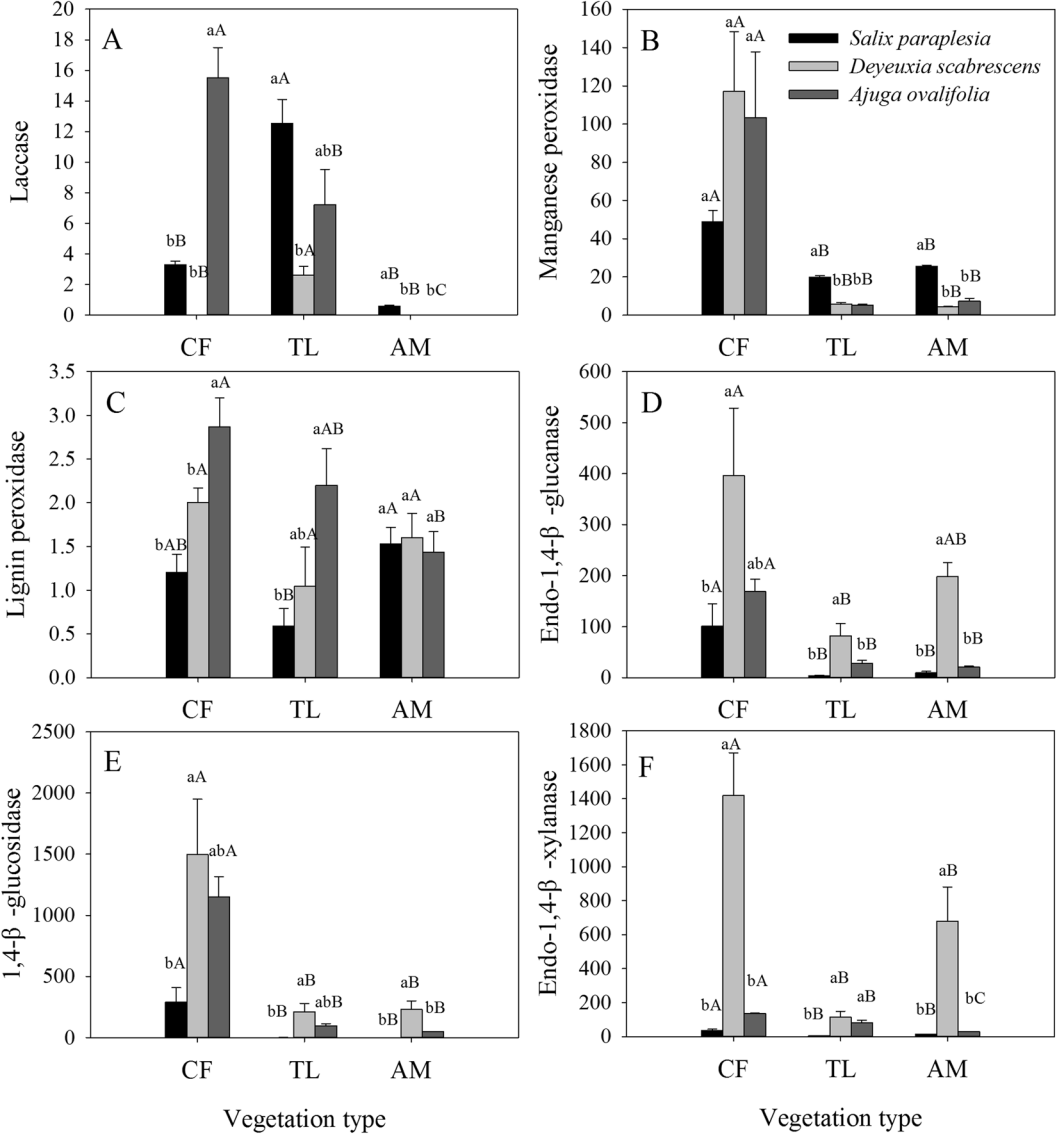
Ligninolytic and cellulolytic enzyme activities (nmol min^−1^ per g C) of *Salix paraplesia* (SP), *Deyeuxia scabrescens* (DS), and *Ajuga ovalifolia* (AO) leaf litter. AM, alpine meadow; TL, tree line; CF, coniferous forest. Different lowercase letters indicate significant differences (P < 0.05) among different litter types within the same vegetation type, and different uppercase letters indicate significant differences (P < 0.05) among vegetation types within the same litter type. The data are the means ± SE (n = 3).

The Lac activities of SP and DS were higher at the tree line than in the coniferous forest and alpine meadow. The Lac activity of AO and the MnP activity of the three species decreased from the coniferous forest to the alpine meadow (Fig. 4A,B), and the MnP activity of SP at the tree line and in the alpine meadow was approximately 3 times higher than that of each of DS and AO (Fig. 4B). The LiP activity of AO was higher than that in SP and DS in the coniferous forest and at the tree line (Fig. 4C). Overall, the EG, βG, and EX activities were higher in the coniferous forest than at the tree line or in the alpine meadow, and the EG, βG, and EX activities of DS were at least twice as high as those of SP and AO except for βG activity in the coniferous forest (Fig. 4D–F).

### Effects of vegetation type and litter type on mass loss, element release and enzyme activities

**Figure 5:**
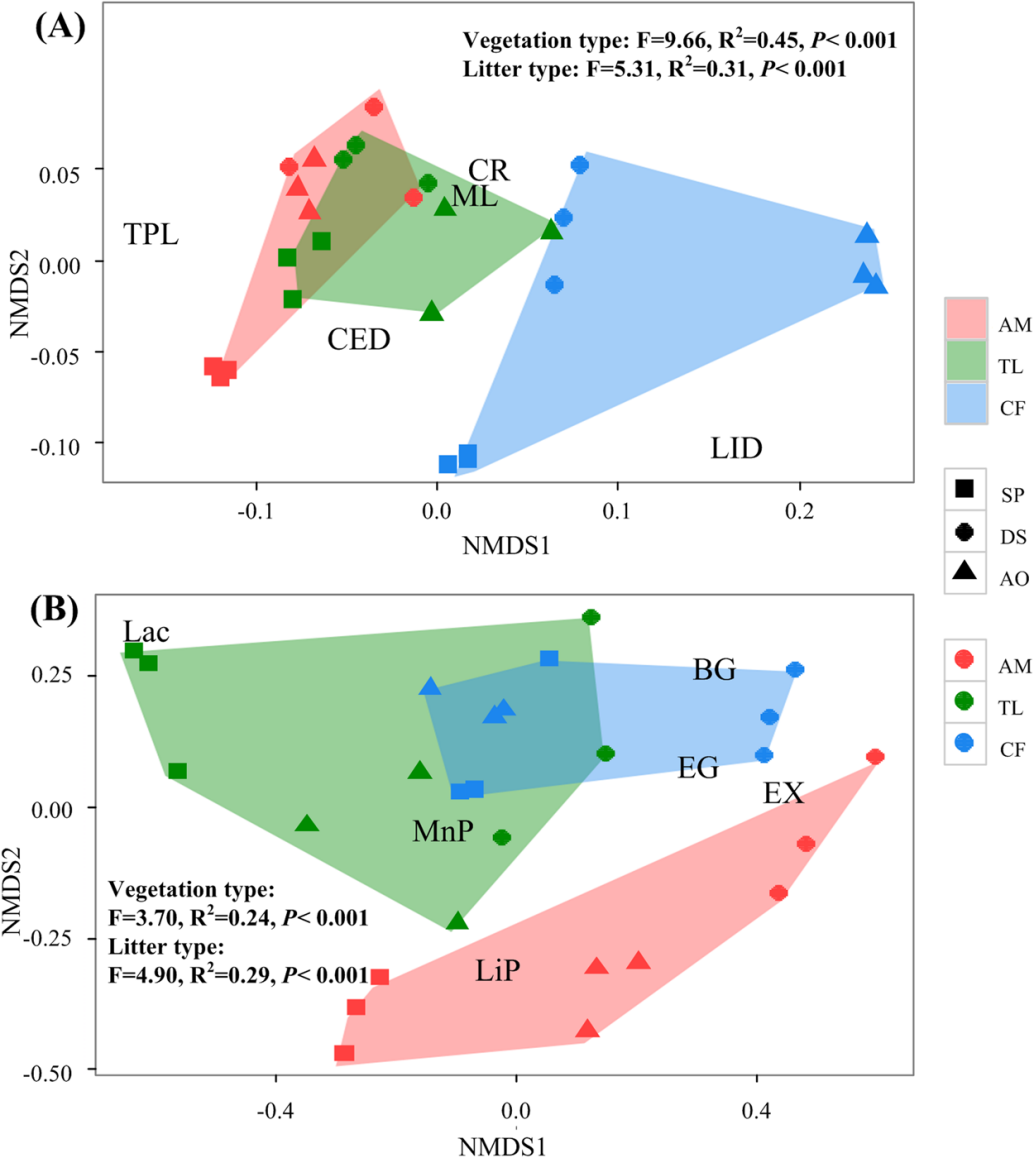
Non-metric multidimensional scaling (NMDS) with Bray-Curtis distance analysis of mass loss, element release (%) (**A**) and enzyme activities (**B**) of leaf litters. NMDS1 shows the effect of vegetation type, and NMDS2 shows the effect of litter type. AM, alpine meadow; TL, tree line; CF, coniferous forest; SP, *Salix paraplesia*; DS, *Deyeuxia scabrescens*; AO, *Ajuga ovalifolia*; ML, mass loss (%); CR, carbon release (%); CED, cellulose degradation (%); LID, lignin degradation (%); TPL, total phenol loss (%); Lac, laccase; MnP, manganese peroxidase; LiP, lignin peroxidase; EG, endo-1,4-β-glucanase; βG, 1,4-β-glucosidase; EX, endo-1,4-β-xylanase.

**Figure 6:**
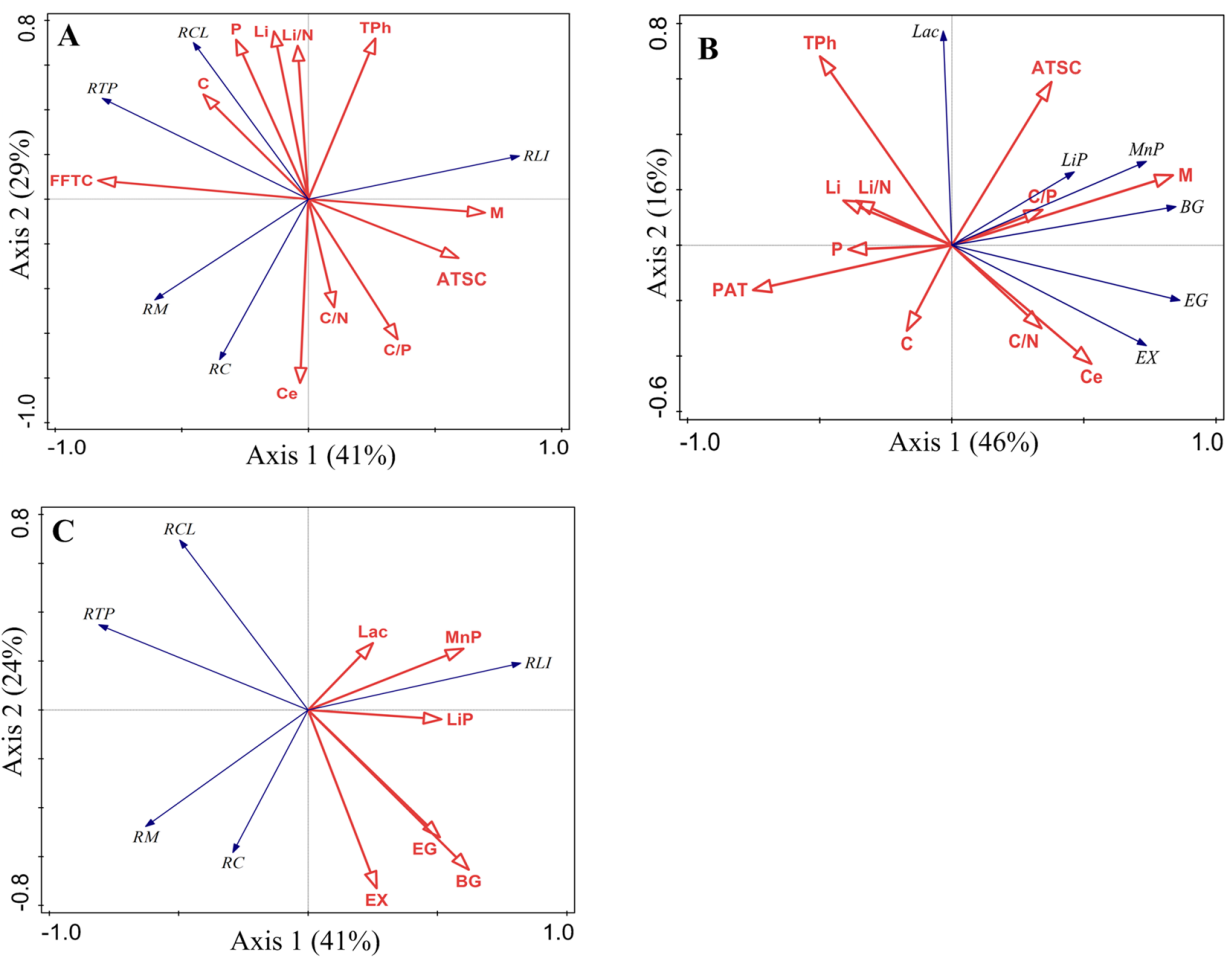
Redundancy analysis of the mass loss, element release (%) (**A**,**C**) and enzyme activities (**B**) of leaf litters. C, carbon concentration; N, nitrogen concentration; P, phosphorus concentration; Ce, cellulose concentration; Li, lignin concentration; TPh, total phenol concentration; M, moisture content of the litter; AT, average temperature; FFTC, frequency of the freeze-thaw cycle; PAT, positive accumulated temperature; NAT, negative accumulated temperature; ATSC, average thickness of the snow cover; ML, mass loss (%); CR, carbon release (%); CED, cellulose degradation (%); LID, lignin degradation (%); TPL, total phenol loss (%); Lac, laccase; MnP, manganese peroxidase; LiP, lignin peroxidase; EG, endo-1,4-β-glucanase; βG, 1,4-β-glucosidase; EX, endo-1,4-β-xylanase.

The two-way ANOVA and NMDS analysis revealed that vegetation type and litter type had significant effects on mass loss (P < 0.05), element release (P < 0.01) and enzyme activities (P < 0.05) but not on C release (P = 0.095) or MnP (P = 0.733) (Table 3; Fig. 5). The RDA showed that 41% of the variation in overall litter decomposition was explained by axis 1, which was mainly related to microclimatic factors, and that 29% of the variation was explained by axis 2, which was mainly related to litter quality. Furthermore, the explanatory power of microclimatic factors (53%) was higher than that of litter quality (28%) in terms of explaining the variation in overall litter decomposition. Among the litter quality and microclimatic variables, FFTC (30%) was the strongest explanatory factor, and mass loss, lignin degradation and total phenol loss were mainly controlled by FFTC, moisture content and ATSC. The release of C and cellulose degradation, which were mainly related to axis 2, were mainly controlled by the concentration of cellulose and C/P (Fig. 6A and Table 4).

**Table 3:** Effects of vegetation type, litter type, and their interactions on mass loss, element release and enzyme activities of leaf litter.

Factors	Vegetation type (df = 2)		Litter type (df = 2)		Vegetation type × litter type (df = 4)	
	F value	P value	F value	P value	F value	P value
Mass loss (%)	4.726	<0.050	8.030	<0.010	2.357	0.092
Carbon release (%)	2.697	0.095	12.311	<0.001	4.603	<0.050
Cellulose degradation (%)	71.223	<0.001	229.126	<0.001	26.57	<0.001
Lignin degradation (%)	149.242	<0.001	35.498	<0.001	11.911	<0.001
Total phenol loss (%)	74.491	<0.001	36.285	<0.001	6.667	<0.010
Laccase	34.054	<0.001	26.397	<0.001	21.043	<0.001
Manganese peroxidase	25.263	<0.001	0.383	0.687	2.907	0.051
Lignin peroxidase	5.158	<0.050	10.096	<0.010	3.218	<0.050
Endo-1,4-β-glucanase	11.763	<0.010	12.375	<0.001	1.428	0.265
1,4-β-glucosidase	27.403	<0.001	8.092	<0.010	3.198	<0.050
Endo-1,4-β-xylanase	13.892	<0.001	40.325	<0.001	11.279	<0.001

**Table 4:** Summary statistics of the redundancy analysis describing the amount of variation (%) in mass loss and element release of leaf litter explained by litter quality and environmental factors and the correlation coefficients between the variables and the axis.

Variable	Simple term effects (% explained)	Conditional term effects (% explained)	Correlation coefficient with axis	
			Axis 1	Axis 2
Litter quality				
(explained 28%^**^ of the total variation)				
C	16^*^	ns	−0.395	0.429
N	13^*^	ns	−0.159	0.534
P	20^**^	ns	−0.272	0.653
C/N	ns	ns	0.097	−0.443
C/P	19^**^	14^**^	0.336	−0.574
Ce	20^**^	20^**^	−0.031	−0.753
Li	18^**^	ns	−0.129	0.688
Li/N	14^*^	ns	−0.040	0.629
TPh	18^**^	ns	0.252	0.659
Environmental factors				
(explained 53%^**^ of the total variation)				
M	22^**^	6^**^	0.664	−0.056
AT	29^**^	ns	−0.784	−0.028
FFTC	30^**^	30^**^	−0.790	0.076
PAT	23^**^	ns	−0.682	−0.136
NAT	29^**^	ns	−0.786	0.084
ATSC	17^**^	5^**^	0.565	−0.242

Significance of the effects: ns, not significant; *P < 0.05; **P < 0.01. Simple term effects show the amount of variation explained by each variable (equivalent to the explanatory power of each individual variable using constrained ordination analysis). Conditional term effects show the increase in the explanatory power after adding a new variable based on the order of explanation. C, carbon concentration; N, nitrogen concentration; P, phosphorus concentration; Ce, cellulose concentration; Li, lignin concentration; TPh, total phenol concentration; M, moisture content of the litter; AT, average daily temperature; FFTC, frequency of the freeze-thaw cycle; PAT, positive accumulated temperature; NAT, negative accumulated temperature; ATSC, average thickness of the snow cover; (n = 27).

Axes 1 and 2 explained 46% and 16%, respectively, of the variation in enzyme activities, and microclimatic factors explained more of the variation (48%) than did litter quality (17%). Moisture content (34%) was the strongest explanatory factor, and the total phenol, cellulose and lignin concentrations were significant explanatory factors related to litter quality (Fig. 6B and Table 5). The βG, EG and EX enzyme activities were positively related to cellulose concentration (Fig. 6B), and the total phenol concentration was positively related to Lac and LiP activities and negatively related to EG and EX activities.

**Table 5:** Summary statistics of the redundancy analysis describing the amount of the variation (%) in enzyme activities explained by litter quality and environmental factors and the correlation coefficients between the variables and the axis.

Variable	Simple term effects (% explained)	Conditional term effects (% explained)	Correlation coefficient with axis	
			Axis 1	Axis 2
Litter quality				
(explained 17%** of the total variation)				
C	ns	ns	−0.153	−0.256
N	ns	ns	−0.321	0.186
P	ns	ns	−0.353	−0.013
C/N	ns	ns	0.306	−0.249
C/P	ns	7**	0.310	0.106
Ce	17**	ns	0.477	−0.355
Li	10**	ns	−0.371	0.133
Li/N	ns	ns	−0.328	0.133
TPh	19**	17**	−0.450	0.565
Environmental factors				
(explained 48% ** of the total variation)				
M	34**	34**	0.755	0.209
AT	30**	ns	−0.700	−0.290
F FTC	27**	ns	−0.64	−0.405
PAT	27**	ns	−0.678	−0.134
NAT	27**	ns	−0.632	−0.412
ATSC	13**	5*	0.340	0.489

Significance of the effects: ns, not significant; *P < 0.05; **P < 0.01. Simple term effects show the amount of variation explained by each variable (equivalent to the explanatory power of each individual variable using constrained ordination analysis). Conditional term effects show the increase in explanatory power after adding a new variable based on the order of explanation. C, carbon concentration; N, nitrogen concentration; P, phosphorus concentration; Ce, cellulose concentration; Li, lignin concentration; TPh, total phenol concentration; M, moisture content of the litter; AT, average temperature; FFTC, frequency of the freeze-thaw cycle; PAT, positive accumulated temperature; NAT, negative accumulated temperature; ATSC, average thickness of the snow cover; (n = 27).

### Effects of enzyme activities on litter mass loss and element release

The RDA showed that the variation in litter mass loss and element release could be affected by enzyme activities. The explanatory power of cellulolytic enzyme activities (48%) was higher than that of ligninolytic enzyme activities (29%) in terms of the variation in litter mass loss and element release. Among the enzyme variables, βG activity (27%) was the strongest explanatory factor (Fig. 6C and Table 6).

**Table 6:** Summary statistics of the redundancy analysis describing the amount of the variation (%) in the mass loss and element release of leaf litter explained by enzyme activities and the correlation coefficients between the variables and the axis.

Variable	Simple term effects (% explained)	Conditional term effects (% explained)	Correlation coefficient with axis	
			Axis 1	Axis 2
Ligninolytic enzyme activities				
(explained 29%** of the total variation)				
Lac	ns	6*	0.239	0.231
MnP	20**	19**	0.572	0.212
LiP	11*	10**	0.491	−0.032
Cellulolytic enzyme activities				
(explained 48%** of the total variation)				
EG	18**	6*	0.485	−0.440
βG	27**	27**	0.592	−0.552
EX	16**	9*	0.253	−0.617

Significance of the effects: ns, not significant; *P < 0.05; **P < 0.01. Simple term effects show the amount of variation explained by each variable (equivalent to the explanatory power of each individual variable using constrained ordination analysis). Conditional term effects show the increase in the explanatory power after adding a new variable based on the order of explanation. Lac, laccase; MnP, manganese peroxidase; LiP, lignin peroxidase; EG, endo-1,4-β-glucanase; βG, 1,4-β-glucosidase; EX, endo-1,4-β-xylanase; (n = 27).

## Discussion

The findings of the present study demonstrated that both microclimate and litter quality had significant effects on overall litter decomposition. Moreover, microclimatic factors played a more important role than did litter quality in explaining the variation in overall litter decomposition at the early decomposition stage. Margesin *et al*.^23^ found that the decomposition of pine needle litter was lower at higher-elevation sites than at lower-elevation sites, whereas no consistent trend of mass loss of the three litter types along the ecotone was detected in the present study. Drewnik^46^ found that cellulose degradation decreased with elevation within a given vegetation zone in a montane-alpine ecotone, which is consistent with our results that lower cellulose degradation in SP and AO litter was observed at higher elevation. During the snow-covered season, the snowpack gradient generates heterogeneity in temperature and moisture^47^. Deep snow cover acts as an insulating layer, whereas shallow and variable snow cover leads to lower soil temperatures and a higher FFTC during the winter^29^. In our study, FFTC was the principal factor determining lignin degradation, which was higher in the environments with greater FFTC, i.e., the tree line or alpine meadow. These results support the view that the decomposition of recalcitrant components is facilitated by freezing events^48,49^. On the one hand, freezing events can promote the physical breakdown of organic matter, on the other hand, thawing events can intensify the leaching of soluble compounds in litter^48,49^. The total phenol loss from all three litter types in the coniferous forest was higher than in the tree line or alpine meadow and was associated with higher moisture content and ATSC. This can be explained by the rapid loss of the labile portion such as polyphenols through hydraulic leaching^50^. It has been shown that lignin usually exhibits accumulation, whereas cellulose may either lose or accumulate in the early period of decomposition^2^. In the present study, the cellulose of SP in the alpine forest-tundra ecotone and the lignin of the three litter types in the coniferous forest exhibited absolute accumulation, which is consistent with the observation in Berg and Mcclaugherty^2^ and Li *et al*.^51^. The acid detergent lignin which was determined by the Van Soest method is composed of both lignin and other acid resistant compounds^33,52^. Hence, one possible explanation for the absolute increase in lignin might be an increase in lignin-like compounds originating from microbial products^51,53^. The effects of litter affinity, which has been termed home-field advantage (HFA, decomposition is accelerated in its home environment), have been reported in previous studies^54,55^, but in our study, the three litter types did not exhibit HFA, possibly due to the selection of different plant functional types and marked variations in the microclimatic conditions along the ecotone.

Our results clearly demonstrated that most ligninolytic and cellulolytic enzyme activities were affected by vegetation type and litter type. Moreover, the effects of microclimatic factors were greater than those of litter quality on the variation in enzyme activities at the early decomposition stage. In particular, moisture content was the strongest explanatory factor for the variation in enzyme activities. It has been demonstrated that fungi are the main producers of extracellular enzymes associated with the degradation of lignin and cellulose^56,57^, particularly during the initial decomposition stages^58^. Soil moisture can directly promote enzyme-catalysed processes^59,60^ as well as indirectly alter enzyme activity by influencing the microbial biomass^60^; for instance, positive correlations have been observed between soil moisture content and fungal biomass^9^. Higher enzyme activities in the litter were correlated with higher fungal biomass; presumably, the fungi were the main source of the enzymes^22^. Air temperature decreases with elevation^61^; however, there was a temperature inversion phenomenon along the forest-tundra ecotone^31^, with the average decomposition temperature of the alpine meadow being higher than that of the coniferous forest despite the alpine meadow’s higher elevation. Furthermore, although a previous study predicted that enzyme activity should substantially increase with increasing temperature^9^, the lignocellulolytic enzyme activities in this study did not increase with temperature. This result might be due to the influence of other variables (such as moisture, solar radiation, and snow cover) that altered the effect of temperature on enzyme activity during litter decomposition.

Both litter type and quality can strongly influence extracellular enzyme activity^10,62^. Differences in initial litter quality, such as the relative proportions of lignin and cellulose, can lead to differences in enzyme activity^11^, and the initial cellulose concentration of DS was higher than that of SP and AO (Table 2), which is consistent with the highest cellulolytic enzyme (EG, βG, and EX) activities being found for DS. In our study, cellulolytic enzyme activities were generally related to the cellulose concentration (Fig. 6B and Table 5), which is consistent with the results of Kanazawa and Miyashita^63^, Linkins *et al*.^64^, and Allison and Vitousek^65^. Interestingly, the ligninolytic enzyme activities rather than the cellulolytic enzyme activities were positively correlated with total phenols at the early decomposition stage in our study. This result is consistent with the opposing responses of the oxidative enzyme and hydrolytic enzyme activities to litter quality (e.g., soluble phenolic compounds, plant litter lignin content and N availability) as identified through principal components and multidimensional scaling analyses of soil and litter enzyme activity^66^. In our study, the correlation between extracellular enzyme activities and the C and N concentrations were not significant, which is inconsistent with the results reported by Schnecker *et al*.^67^.

The observed ligninolytic and cellulolytic enzyme activities partially accounted for the variation in overall litter decomposition, which suggested that microbial activity played a vital role during litter decomposition in the snow-covered season^30,68,69^. In our study, the explanatory power of cellulolytic enzyme activities was higher than that of ligninolytic enzyme activities in terms of the variation in litter decomposition. This result could be explained by cellulose being preferentially degraded to yield glucose during the early stages of plant litter decomposition^1,5^. The final step in cellulose depolymerisation, namely, the hydrolysis of cellobiose to glucose, is catalysed by βG, which is the most commonly measured cellulolytic enzyme in ecological studies^5^. Among enzyme variables, the activity of βG was the factor explaining most of the variation in the overall litter decomposition (Fig. 6C and Table 6).

In summary, the overall litter decomposition and cellulolytic and ligninolytic enzyme activities were significantly influenced by microclimate variables and litter type during the early litter decomposition stage. Microclimatic factors were found to be stronger predictors than litter quality of the variations in litter decomposition and lignocellulolytic activity. Interestingly, FFTC and moisture were the predominant microclimate factors. In addition, the explanatory power of cellulolytic enzyme activities was higher than that of ligninolytic enzyme activities at the early stage of litter decomposition. In contrast to the pattern observed for lignin degradation, the total phenol loss, MnP and cellulolytic enzyme activities of the three litter species were higher in the coniferous forest than at the treeline or in the alpine meadow. The higher cellulose degradation was consistent with the higher cellulose concentration and cellulolytic enzyme activities in the litter. Therefore, under climate change, the direction and magnitude of changes in microclimatic factors and litter quality warrant study in alpine ecosystems.

## Data Availability

The datasets generated and/or analysed in the current study are available from the corresponding author upon reasonable request.
